# Spinal Reflex Recovery after Dorsal Rhizotomy and Repair with Platelet-Rich Plasma (PRP) Gel Combined with Bioengineered Human Embryonic Stem Cells (hESCs)

**DOI:** 10.1155/2020/8834360

**Published:** 2020-10-29

**Authors:** Mateus Vidigal de Castro, Moníze Valéria Ramos da Silva, Gabriela Bortolança Chiarotto, Maria Helena Andrade Santana, Ângela Cristina Malheiros Luzo, Sergiy Kyrylenko, Alexandre Leite Rodrigues de Oliveira

**Affiliations:** ^1^Department of Structural and Functional Biology, Institute of Biology, University of Campinas, Campinas, São Paulo, Brazil; ^2^University Center of Herminio Ometto Foundation, Araras, São Paulo, Brazil; ^3^Department of Engineering of Materials and Bioprocesses, School of Chemical Engineering, University of Campinas, Campinas, São Paulo, Brazil; ^4^Haematology and Hemotherapy Center, Umbilical Cord Blood Bank, University of Campinas, Campinas, São Paulo, Brazil; ^5^Biomaterials Research Group, Center for Collective Use of Scientific Equipment, Medical Institute of Sumy State University, Sumy, Ukraine

## Abstract

Dorsal root rhizotomy (DRZ) is currently considered an untreatable injury, resulting in the loss of sensitive function and usually leading to neuropathic pain. In this context, we recently proposed a new surgical approach to treat DRZ that uses platelet-rich plasma (PRP) gel to restore the spinal reflex. Success was correlated with the reentry of primary afferents into the spinal cord. Here, aiming to enhance previous results, cell therapy with bioengineered human embryonic stem cells (hESCs) to overexpress fibroblast growth factor 2 (FGF2) was combined with PRP. For these experiments, adult female rats were submitted to a unilateral rhizotomy of the lumbar spinal dorsal roots, which was followed by root repair with PRP gel with or without bioengineered hESCs. One week after DRZ, the spinal cords were processed to evaluate changes in the glial response (GFAP and Iba-1) and excitatory synaptic circuits (VGLUT1) by immunofluorescence. Eight weeks postsurgery, the lumbar intumescences were processed for analysis of the repaired microenvironment by transmission electron microscopy. Spinal reflex recovery was evaluated by the electronic Von Frey method for eight weeks. The transcript levels for human FGF2 were over 37-fold higher in the induced hESCs than in the noninduced and the wildtype counterparts. Altogether, the results indicate that the combination of hESCs with PRP gel promoted substantial and prominent axonal regeneration processes after DRZ. Thus, the repair of dorsal roots, if done appropriately, may be considered an approach to regain sensory-motor function after dorsal root axotomy.

## 1. Introduction

One of the most severe lesions that affects the brachial plexus is the rupture of nerve roots in their sites of connection to the spinal cord. Although there is still no treatment capable of recovering all lost functions to victims due to the well-known limited regeneration ability of the central nervous system (CNS), numerous approaches have been tested for promoting functional recovery, from advanced surgical techniques to pharmacological techniques, to overcome this situation [1].

The physical disconnection of the dorsal roots from the surface of the spinal cord, as occurs in dorsal rhizotomy (DRZ), results in a series of cellular and molecular events that directly affect the sensory pathways [2]. In this case, fibers with different sensory modalities are unable to reach the spinal cord, leading to significant changes in the spinal circuits [3, 4] that are also reflected in motor behavior [2]. The major challenge for recovery techniques is how to allow the primary afferent fibers to enter the spinal cord environment and form synapses in specific layers [5], which would then reestablish the sensory circuits and pathways [6].

In addition to the extensive degeneration of primary afferent fibers that enter the spinal cord through the dorsal root, a rapid inflammatory reaction characterized by the proliferation, recruitment, and activation of cells of the nervous tissue and the immune system occurs in response to lesions [7]. In this way, the reactive astrocytes and microglia form a dense nonpermissible wall by joining communicating junctions (the so-called glial scar), creating a physical barrier to axonal growth [8]. These cells also produce many inflammatory mediators that can have detrimental effects on neuronal survival and axonal growth, playing an important role in inhibiting the regeneration of damaged central nervous axons [9].

Thus, therapies that involve the release of proregenerative molecules, blocking of inhibitory molecules, or providing growth permissive substrates have been tested and addressed to augment CNS regeneration with the goal of improving DRZ management [10] for patients with DRZ. Stem cell therapy is one of the most attractive strategies that has been investigated, and it has already shown positive results regarding regeneration and functional recovery in some experimental models of lesions and diseases that affect the CNS [11]; these positive impacts are due to multiple advantages that stem cell therapy can bring to the lesion microenvironment.

The current knowledge suggests that grafted cells can play their supporting role by delivering trophic factors [12]. It was shown that the effects of cell therapy are mediated by the secretion of growth factors and/or cytokines that reduce the process of apoptosis and neuronal inflammation and also stimulate endogenous regenerative processes, remyelination, and neural plasticity [12, 13].

In light of the inhibitory and limiting factors of central regeneration, in this study, we proposed the use of bioengineered human embryonic stem cells (hESCs) that inductively overexpress human growth factor 2 (FGF2), also known as basic fibroblast growth factor, which plays an important role in axonal regeneration after spinal cord injuries [14, 15]. Here, the cells were delivered to the injury site (dorsal rhizotomy in a rat model) in an organic 3D matrix formed by platelet-rich plasma (PRP) gel and were used as a source of FGF2 and other proregenerative factors. In our previous work, we standardized the use of PRP gel to treat DRZ, which reconnected the roots with the spinal surface, and enabled axonal regeneration. Here, the PRP gel was also used as a scaffold for hESCs, to potentialize the process of axon regeneration.

The gel is formed by the combination of platelet-rich plasma (PRP), serum containing thrombin, and calcium chloride in the proper volumetric proportions. In turn, PRP, also known as autologous platelet plasma, platelet-enriched plasma, or even platelet concentrate [16], is a high concentration of platelets in a small volume of plasma that originates from the processing of autologous whole blood by centrifugation [17]. Once thrombin and calcium chloride are added to the platelets, PRP forms a gel-like structure that acts as a biological glue. This chain reaction forms a matrix of bioactive fibrin with hemostatic and adhesion properties, mimicking the final steps of the coagulation cascade [18].

Interestingly, platelets, the main component of PRP, actively participate in the blood clotting process through the formation of the blood clot [19], and they can also produce and release a wide range of cell signaling molecules, such as cytokines, trophic factors, and growth factors that perform many biological actions [20]. Once PRP is applied on the lesion site as a liquid-to-gel scaffold, tissue fibrinolysis breaks the fibrin down, releasing the cocktail of factors with mitogenic and chemotactic properties from the platelet *α*-granules, which can be beneficial to the injured microenvironment.

Therefore, based on the effectiveness of the formed glue to reconnect spinal roots and the multiple properties of platelets in the biological microenvironment, as well as the combined advantages of cytotherapy with stem cells in regenerative medicine, we proposed the use of platelet-rich plasma (PRP) gel as a biological glue and as an organic scaffold for bioengineered hESCs for the treatment of DRZ. In this way, we hope that this approach opens a new and alternative front for the treatment of spinal root injuries and contributes to the future clinical use of this type of therapy.

## 2. Materials and Methods

### 2.1. Experimental Design

In the present study, adult female Lewis rats (~200 g, 8-10 weeks old) were subjected to a unilateral rhizotomy (DRZ) of the L4-L6 dorsal roots and then were divided into the following groups: (a) unlesioned/control, (b) DRZ without repair (DRZ), (c) DRZ followed by root repair with platelet-rich plasma gel (PRP), and (d) DRZ followed by root repair with PRP and bioengineered human embryonic stem cells (hESCs). The animals were obtained from the Multidisciplinary Center for Biological Investigation (CEMIB/UNICAMP) and were housed under a 12-hour light/dark cycle with free access to food and water. All procedures were performed in accordance with the ethical principles regulated by the National Council of Animal Experimentation (CONCEA) and with the approval of the Ethics Committee on Animal Experimentation of the University of Campinas (CEUA/UNICAMP, protocol no. 4169-1B) and the Ethics Committee of the School of Medical Sciences of Unicamp (Campinas; CAAE 78627717.0.0000.5404).

The animals were killed 1 week (acute phase) and 8 weeks (chronic phase) after establishing lesions, and their lumbar spinal cords were processed for immunofluorescence and electron microscopy analysis. Additionally, rats underwent 8 weeks of behavioral testing (electronic von-Frey), which was performed weekly (Supplementary Figure S1).

### 2.2. Human Platelet-Rich Plasma (PRP) Gel

Human platelet-rich plasma gel was prepared as previously described [21]. Briefly, PRP gel was obtained by mixing platelet-rich plasma with serum thrombin, both of which were isolated from human blood subjected to centrifugation steps and 10% calcium chloride treatment (complete methods in the Supplementary Material and Methods).

### 2.3. Bioengineered Human Embryonic Stem Cells (hESCs)

The cells used in this study were the human embryonic stem cell line CCTL12, derived at the Masaryk University in Brno, Czech Republic. The cells were bioengineered for inducible overexpression of the 18 kDa isoform of human fibroblast growth factor 2 (FGF2) and then were cultivated for use in experiments with dorsal rhizotomy (DRZ) in rats. Cells were engrafted directly at the lesion site as described below, in the PRP scaffold.

#### 2.3.1. Establishment of Stable Transgenic Cells

Methods for establishing stable transgenic cells were described in detail elsewhere [22]. Briefly, EF1a Tet-On 3G system (Clontech® Laboratories, Cat#631167) was used for the derivation of stable clones with inducible overexpression. Transfection was performed with the FuGENE HD Transfection Reagent (Roche, Switzerland). Positive clones for the Tet-On presence were transfected with the pTe106 target vector (GenBank accession number: KX844812), which contains the FGF2-GFP fusion ORF, where green fluorescent protein (GFP) was originated from pEGFP-N1 vector (Clontech® Laboratories, Cat#6085-1). Vectors for stable transfections were used in a linearized form obtained by preparative PCR. Selection was performed with G-418 at 140 *μ*g/mL and blasticidin at 1.2 *μ*g/mL, according to the carefully predetermined selection profiles. Cells were transfected, detached 24 hours posttransfection, and plated into 6-well plates in serial dilutions. Selection was carried out for two weeks with regular changes of medium as described. Induction of the transgene was achieved by treatment with 1 *μ*g/mL doxycycline for 48 h. The resulting stable clone E12-1-1 (inducibly overexpressing human FGF2 fused with the GFP), which underwent dual selection, was used in further experiments. The cell karyotypes were confirmed by the Institut für Humangenetik und Anthropologie, Jena, Germany.

#### 2.3.2. Culturing

**Figure 1:**
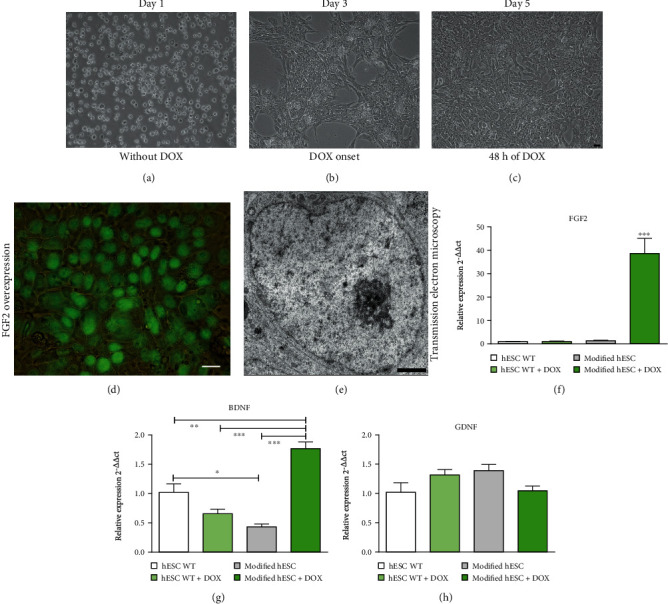
(a–c) Cultivation of bioengineered human embryonic stem cell (hESC) clones over time. The formation of the cell monolayer (confluence) was observed on the fifth day (c). The cells were attached to a matrix (Matrigel) and expanded rapidly in their own medium, and they formed colonies and/or aggregates. Bright field. Scale bar = 10 *μ*m. (d) Clones were induced to overexpress FGF2 at 48 hours after doxycycline administration. Practically all cells were fluorescent green (GFP+), which indicates that hESCs were activated by DOX and then overexpressed FGF2. Phase contrast. Scale bar = 25 *μ*m. (e) A single bioengineered hESC was observed under electron microscopy, enabling visualization of the large nucleus, very evident nucleolus, and euchromatin, indicating high metabolic activity. Scale bar = 1 *μ*m. (f–h) Relative expression of basic fibroblast growth factor (FGF2), brain-derived neurotrophic factor (BDNF), and glial cell line-derived neurotrophic factor (GDNF) mRNA *in vitro*. (f) The transcript levels for FGF2 were over 35-fold higher when modified hESCs were induced by doxycycline. Modified hESCs not treated with doxycycline and wildtype cells treated or not treated with doxycycline presented nearly undetectable expression of FGF2. (g) Compared with other groups, the transcript levels for BDNF were significantly higher when modified hESCs were induced by doxycycline. (h) The transcript levels for GDNF were not significantly different between groups. Mean ± SEM. ^∗∗∗^*p* < 0.001. hESCs: human embryonic stem cells; WT: wildtype; DOX: doxycycline.

Matrigel-covered plates (Corning Life Sciences, USA) were used for culturing bioengineered hESCs in monolayers as previously described [23]. The cells were kept at 37°C under a 5% CO_2_ atmosphere in mTeSR™1 medium (STEMCELL Technologies™; code 85850), until they established a monolayer (Figure 1). Doxycycline (DOX) was added to the medium at a final concentration of 1 *μ*g/mL, 48 hours before detachment; DOX induced FGF2 overexpression in the bioengineered hESCs *in vitro*. After that, the cells were detached by TrypLE™ Express (Gibco**®** by Life Technologies; code 12605-028), collected, washed, and counted in a Neubauer chamber.

#### 2.3.3. Engrafting

Immediately following DRZ, 3 × 10^5^ hES cells resuspended in 5 *μ*L of mTeSR™1 medium (STEMCELL Technologies™; code 85850) were engrafted directly at the lesion site and mixed together with the PRP matrix/scaffold before its polymerization. To induce overexpression of FGF2 in hESCs *in vivo*, DOX was given to animals in combination with food pellets *ad libitum* for the whole duration of the experiment, at a concentration of 625 mg of DOX per kg of food pellets, as described in [24]. Induction was confirmed by GFP expression in the hESCs.

#### 2.3.4. Phenotyping

Characterization of the pluripotency and differentiation state of bioengineered human embryonic stem cells (hESCs) was performed by multicolor flow cytometry assays using a BD Stemflow™ Human and Mouse Pluripotent Stem Cell Analysis Kit in accordance with the manufacturer's instructions. The kit contained 3 fluorochrome-conjugated antibodies for distinguishing pluripotent cells from differentiated cells: (1) Oct3/4 (octamer-binding transcription factor 4), also known as POU5F1, a transcription factor expressed in pluripotent stem cells; (2) SSEA-1 (stage-specific embryonic antigen-1), a differentiation marker in embryonic cells; and (3) SSEA-4 (stage-specific embryonic antigen-4), which is a pluripotency marker in embryonic cells.

#### 2.3.5. Gene Expression

The relative mRNA levels of FGF2, BDNF, and GDNF genes in the *in vitro* bioengineered human embryonic stem cells were evaluated by qRT-PCR. These cells were divided into 4 groups: wildtype hESCs; wildtype hESCs + DOX; bioengineered hESCs; and bioengineered hESCs + DOX. Each group was analyzed in triplicates of 1.8 × 10^−6^ cells. Total RNA was extracted from cells, 48 hours after activation by doxycycline, using an RNeasy Lipid Tissue Mini Kit (cat. no. 74804, Qiagen) according to the manufacturer's instructions. The quantity, quality, and integrity of the RNA samples were determined using a nanophotometer and agarose gel electrophoresis under denaturing conditions. A High-Capacity cDNA Reverse Transcription Kit (Applied Biosystems: 4368814) was employed to convert 2 *μ*g of total RNA into cDNA, which was used in triplicate as a template for PCR reaction. qRT-PCR was performed with an Mx3005P instrument (Agilent Technologies, Santa Clara, CA, USA), with the TaqMan Gene Expression Master Mix (2x) (Life Technologies: PN 4369016) and TaqMan reagents (Table 1) in a volume of 20 *μ*L. The following thermocycling conditions were used: 45 cycles for amplification (95°C for 10 minutes, followed by 95°C for 15 seconds and 60°C for 1 minute).The 2^−ΔΔCt^ method [25] was performed for relative quantification, and GAPDH was used as the housekeeping gene.

### 2.4. Dorsal Rhizotomy and Repair

**Figure 2:**
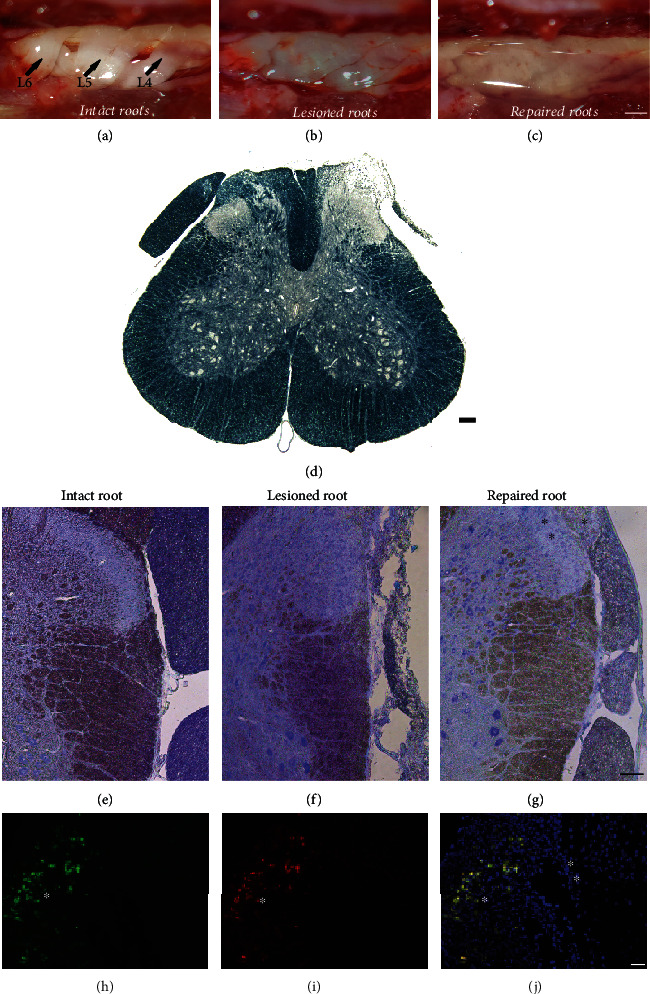
(a–c) Region of lumbar intumescence of an injury in a rat, seen through a surgical magnifying loupe. (a) Intact dorsal roots (L4, L5, and L6). (b) Transected dorsal roots (L4, L5, and L6). (c) PRP gel on the transected dorsal roots (L4, L5, and L6), immediately after its application. Immediately after applying the gel, it started to polymerize. From then on, it was no longer possible to reposition the roots that were transected on the spinal surface. Therefore, the repositioning of the roots after injury must be accurate before applying the gel. It is also possible to observe some hemorrhage after the lesion induction, where it was stopped with the gel. Scale bar = 5 mm. (d) Representative photomicrograph of transverse sections of the spinal cord stained with Sudan black 8 weeks after DRZ. The lesioned ipsilateral root was completely degenerated, as opposed to the contralateral root, which exhibits high integrity. Scale bar = 100 *μ*m. (e–g) Photomicrograph of transverse sections of the hemispinal cord stained with toluidine blue, 8 weeks after the DRZ. (e) The unlesioned root showed a large number of axons. (f) The lesioned root was completely disrupted. (g) The repaired root showed a significant number of axons. Scale bar = 100 *μ*m. (h–j) Bioengineered human embryonic stem cells (hESCs) found in the roots repaired with PRP, two weeks after injury: (h) hESCs overexpressing FGF2 (GFP+), in green; (i) hESCs marked with an antibody specific for human mitochondria (hMito), in red; (j) merge: GFP+ (green) + hMito (red), demonstrating that the engrafted cells are in fact the modified bioengineered hESCs. In blue, nuclear DNA labeling was performed using DAPI. ^∗^Repaired root. ^∗∗^Spinal cord. The cells remained in the replanted root. Scale bar = 20 *μ*m.

Animals were anesthetized by a combination of xylazine chlorhydrate (Anasedan®, 10 mg/kg, Sespo Indústria e Comércio, Paulínia, SP, Brazil) and ketamine hydrochloride (Dopalen®, 50 mg/kg, Sespo Indústria e Comércio, Paulínia, SP, Brazil) and then were subjected to unilateral spinal dorsal rhizotomy of the dorsal roots, which was performed based on the protocols of a previous work [21, 26]. Lesions were generated on the right side of the L4, L5, and L6 lumbar dorsal roots after laminectomy. A longitudinal incision was made to open the dural sac, and the dorsal roots associated with the lumbar intumescence were identified and cut 2 mm from the surface of the spinal cord with microscissors (Figures 2(a)–2(c)).

In the treatment groups, the roots were replaced at the exact point of detachment, on the dorsal surface of the lumbar spinal cord at the lesion site with the aid of the PRP gel (Figure 2(c)). For this procedure, during surgical repair of the lesioned roots, the components to activate PRP were mixed in an Eppendorf© tube at the following concentrations: 30 *μ*L of PRP + 2.625 *μ*L of autologous serum + 0.875 *μ*L of 10%calcium chloride. Then, the mixture was applied at the lesion site using a micropipette immediately after the lesion was created. Then, the lesioned roots were returned to their original sites. Additionally, in the PRP + cell groups, 3 × 10^5^ hESCs were added to the activated PRP, at the moment of repair.

After the surgical procedures, the muscle fascia and skin were sutured in layers. The animals were housed under a 12 h light/dark cycle and controlled temperature with free access to food and water for a period of up to 8 weeks. Tramadol chlorhydrate (Germed Farmacêutica Ltda, Hortolândia, SP, Brazil) was administered after the surgical procedure (20 mg/kg, by gavage) for 5 days (2.5 mg/day, dissolved in drinking water).

### 2.5. Functional Analysis-Reflex Arc Evaluation (Von Frey)

The animals were individually placed in acrylic boxes with a wire grid floor (10 × 10 × 20 cm) with a tilted mirror below them, to provide a view of the hind paws. After habituation, a gradual increasing pressure was applied on the central plantar area of the right hind paw with a 0.5 mm^2^ polypropylene tip coupled to a handheld force transducer (electronic anesthesiometer, EFF 301 by Insight, Ribeirão Preto, Brazil), evoking flexion reflex and paw withdrawal. Pressure intensity was automatically recorded three times for each animal, with an interval of approximately 10 minutes between each measurement. The maximum pressure limit established was 80 g. If the animal did not respond to this intensity of pressure, it was considered to exhibit total paw anesthesia. The reflex paw flinches are shown as the mean ± standard error of the mean (SEM) of the weekly measurements.

### 2.6. Specimen Preparation

Animals were anesthetized by an overdose of a combination of xylazine chlorhydrate (Anasedan®, 10 mg/kg, Sespo Indústria e Comércio, Paulínia, SP, Brazil) and ketamine hydrochloride (Dopalen®, 50 mg/kg, Sespo Indústria e Comércio, Paulínia, SP, Brazil), and the vascular system was rinsed by transcardial perfusion with cold 0.1 M saline phosphate buffer (PBS; pH 7.38), which was followed by a rinse with a fixative solution.

For immunofluorescence analysis, the rats were killed 1 or 8 weeks after DRZ. After perfusion with PBS, the tissues were fixed by vascular perfusion with 4% paraformaldehyde in phosphate buffer (PB 0.1 M, pH 7.38). The lesioned region of the spinal cord was exposed, dissected, and postfixed overnight at 4°C in the same fixative solution, and then they were subjected to gradually increased concentrations of sucrose (10%, 20%, and 30% sucrose in 0.1 M sodium phosphate buffer, 24 h in each solution) before freezing. The specimens were embedded into Tissue-Tek® O.C.T. (Sakura Finetek USA, Inc., Torrance, CA USA) and were frozen at -40°C. Transverse sections (12 *μ*m thick) were obtained with a cryostat (Microm HM 525®, Microm International GmbH, Walldorf, Germany), and then they were transferred to gelatin-coated slides and dried at room temperature for 30 minutes; then, they were stored at -20°C until immunolabeling was performed.

For electron microscopy analysis, the rats were killed 8 weeks after DRZ surgery. After perfusion with PBS, the tissues were fixed by vascular perfusion with 2.5% glutaraldehyde and 1% paraformaldehyde in phosphate buffer (PB 0.1 M, pH 7.38). The lesioned region of the spinal cord was exposed, dissected, and fixed for 24 hours at 4°C in the same fixative solution. The samples were then trimmed and postfixed in 1% osmium tetroxide solution in phosphate buffer (PB 0.1 M, pH 7.38). After postfixation, the samples were washed in distilled water, dehydrated through treatment with a graded ethanol series and acetone, and then embedded in Durcupan ACM (Fluka, Steinheim, Switzerland).

### 2.7. Immunofluorescence

Transverse sections of the spinal cord were allowed to come to room temperature, washed twice with 0.01 M phosphate buffer (PB) (pH 7.4), blocked with blocking solution for 45 minutes, and incubated with primary antibodies for 4 hours in a humid chamber at room temperature. After rinsing with 0.01 M PB (pH 7.4), samples were incubated with secondary antibodies and then they were washed twice with 0.01 M PB (pH 7.4). Primary and secondary antibodies are indicated in Table 2. Slides were mounted with coverslips on glycerol solution (glycerin and distilled water, 3 : 1) containing 4′,6-diamidine-2′-phenylindole dihydrochloride (DAPI, DNA dye, 1 : 1000), and then were observed under a fluorescence microscope (Leica DM5500 B microscope) coupled with a Leica DFC345 FX camera (Leica Microsystems CMS GmbH). For primary antibodies, the blocking solution was prepared with 3% bovine serum albumin solution in 0.1 M PB (pH 7.38), while the antibody dilution solution (for primary and secondary antibodies) was prepared with 1% BSA and 2% Triton X in 0.1 M PB (pH 7.4).

For quantitative measurements, three representative images of the spinal cord (L4-L6) of each animal were captured at a final magnification of 200x. The integrated density of pixels (IDP), which represents the intensity of labeling, was measured utilizing ImageJ software (version 1.45s, National Institute of Health, USA). The data are represented as the mean ± standard error of the mean (SEM) for each group.

### 2.8. Transmission Electron Microscopy (TEM)

Trimmed blocks were cut in an ultramicrotome (Leica Ultracut UCT Ultramicrotome), and semithin transverse sections (0.5 *μ*m) were obtained and stained with 0.25% toluidine blue for light microscopy observation. Then, ultrathin transverse sections (70 nm) from the L4 to L6 segments were made in the same ultramicrotome (Leica Ultracut UCT Ultramicrotome), collected on formvar-coated single-slot grids, and then contrasted with uranyl acetate (2%) and lead citrate. Finally, the ultrathin transverse sections (70 nm) were examined under a Tecnai G2 Spirit BioTwin (FEI, Eindhoven, The Netherlands) transmission electron microscope, to evaluate structural and morphological changes in the microenvironment and adjacent areas 8 weeks after dorsal rhizotomy (chronic phase) and repair.

### 2.9. Statistical Analysis

Data analysis was performed with GraphPad Prism (version 7.00 for Windows, GraphPad Software, La Jolla, California, USA). Immunofluorescence and qRT-PCR data were evaluated via one-way ANOVA followed by Bonferroni multiple comparisons test. Data from the functional analysis (Von Frey) were evaluated via two-way ANOVA followed by Bonferroni Multiple Comparison Test. The data are presented as the mean ± standard error of mean (SEM) and the differences between groups were considered significant when the *p* value was >0.05 (^∗^), >0.01 (^∗∗^), or >0.001 (^∗∗∗^).

## 3. Results

### 3.1. Dorsal Rhizotomy Overview

After DRZ, many structural and morphological changes in the spinal cord microenvironment and roots could be observed. The lesion had an amplitude that covered a large part of the dorsal columns and funiculus. In this model, 8 weeks after lesion induction, it was possible to report the absence of the ipsilateral dorsal root (L4-L6), which was completely degenerated as a result of the lesion. Also, an intense demyelination and degeneration in the site of injury and adjacent areas was depicted, resulting in a large reduction in sensory fibers. No changes were noticed in the contralateral dorsal root. Additionally, there was a large number of infiltrated cells at the lesion site (which could be seen in the immunofluorescence analyses and were mostly macrophages). Figures 2(d)–2(g) show a spinal cord, 8 weeks after dorsal rhizotomy that demonstrates some of these changes in comparison with a repaired spinal cord.

### 3.2. Characterization of Platelet-Rich Plasma Gel

The three-dimensional mesh (Supplementary Figure S1) formed from the volumetric proportions of PRP, thrombin, and calcium chloride defined here was similar to the medium fibers that were described by Perez et al. [27]. Gel fibers of this caliber allowed the regenerating axons to pass through the gel and reach the spinal cord. In addition, they also served as scaffolds for the cells, where the cells were “trapped” within the fibers of the gel. Thin fibers would not provide structure for the cells, while thick fibers would prevent axons from entering the spine.

### 3.3. Bioengineered hESC Phenotyping by Flow Cytometry

Characterization of the cellular pluripotency and differentiation state of bioengineered human embryonic stem cells (hESCs) was performed by flow cytometry assays using a BD Stemflow™ Human and Mouse Pluripotent Stem Cell Analysis Kit. Approximately 97.9% of the bioengineered hESCs expressed SSEA-4, 60.6% expressed Oct3/4, and 0.1% expressed SSEA-1 (Supplementary Figure S2). Thus, the analysis allowed us to define an undifferentiated pluripotent human embryonic stem cell population. In addition, the sample was a homogeneous population regarding size and granularity. Additionally, isotype controls were used to identify any nonspecific (background) labeling by specific antibodies present in the kit (Supplementary Figure S3).

### 3.4. Gene Expression in Bioengineered hESCs Characterized by qRT-PCR

Relative mRNA levels of the FGF2, BDNF, and GDNF genes in hESCs *in vitro* were evaluated by qRT-PCR. The normalized quantification of the transcripts for FGF2, BDNF, and GDNF is shown in arbitrary units and confirmed that both wildtype and bioengineered cells expressed the genes of interest, regardless of whether they were induced with doxycycline (see Figures 1(f)–1(h)). The degree of FGF2 transcription was approximately two orders of magnitude higher in the induced hESCs than in the noninduced and wildtype counterparts, highlighting that bioengineered hESCs induced with doxycycline overexpressed FGF2. These data demonstrate that hESCs can be bioengineered to produce lines with the properties needed for the nervous regeneration process. Additionally, BDNF gene expression was significantly higher in the bioengineered cell group than it was in the other groups. GDNF gene expression did not show significant differences between groups.

### 3.5. Fate of Bioengineered hESCs after Engrafting

After engraftment of the bioengineered hESCs (in the PRP scaffold) directly at the DRZ site, the site was analyzed two weeks after injury (during which the inducer DOX was supplied to animals *via* food pellets). GFP-positive cells were observed at the injury site, especially in the repaired dorsal root (see Figures 2(h)–2(j)). Immunolabeling of anti-human mitochondria confirmed the presence of human cells.

### 3.6. Reflex Arc Evaluation by Electronic Von Frey

**Figure 3:**
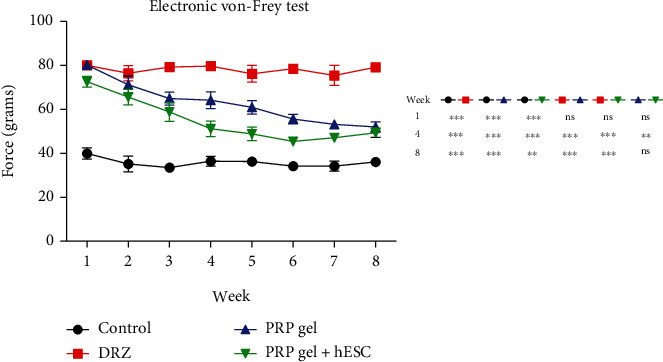
Electronic Von Frey measurements (mean values) obtained from the right hind paw (lesioned). The values are shown as the grams applied to trigger the “flinch” response. Statistical differences among groups are indicated with asterisks in addition to the graph. ^∗^*p* < 0.05; ^∗∗^*p* < 0.01; ^∗∗∗^*p* < 0.001. Only the groups that underwent root repair with the PRP gel demonstrated the recovery of sensitivity to tactile stimulation. Further, the PRP gel + hESC group had the best performance in the whole experiment.

The reflex arc was measured weekly by the electronic Von Frey test. As shown in Figure 3, there were many significant differences between groups during the test. Overall, the PRP gel + hESC group had the best improvements, followed by the group of the PRP gel without cells, while there was no response in the DRZ group. Moreover, one week after causing the lesion, it was observed that animals from the DRZ group presented total anesthesia of the ipsilateral hind paw, exhibiting no flinch response (score 80 grams). This result was consistent over the 8 weeks of analysis. On the other hand, the measures of the animals from the repaired groups gradually improved, indicating partial recovery of the reflex arc over time. Although there was no significant difference between the repair treatment groups in the last week of analysis, at some experimental times, the PRP gel + hESCs group had the best performance, approaching that of the control group. In turn, the animals in the control group (unlesioned) responded to the tactile stimulus (with a flinch response of approximately 35 grams) at all experimental times of analysis. We emphasize that exaggerated reactions that may suggest allodynia or hyperalgesia were not observed in any of the groups.

### 3.7. Immunofluorescence

**Figure 4:**
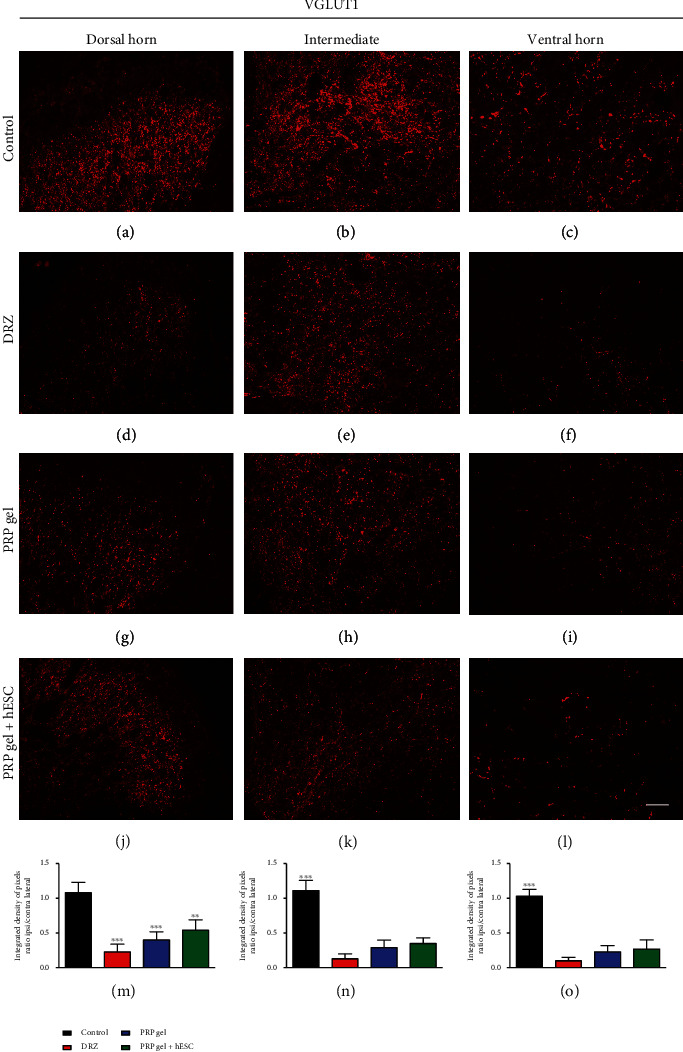
Representative immunofluorescence micrographs of VGLUT1 (glutamatergic synapses) immunolabeling on the ipsilateral side of the spinal cord after rhizotomy and repair, 1 week after lesion induction. A significant decrease in glutamatergic synaptic density was observed following DRZ. However, the “hESC” group showed great improvement in punctate labeling in comparison to the DRZ group. Scale bar = 50 *μ*m.

**Figure 5:**
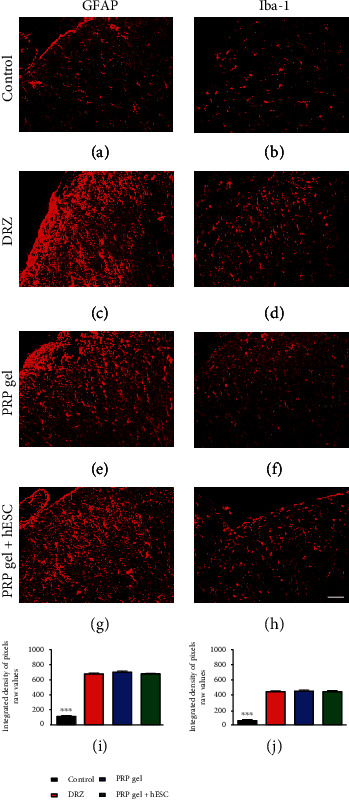
Representative immunofluorescence micrographs of GFAP (astrocytes) and Iba-1 (microglia) immunolabeling on the ipsilateral side of the spinal dorsal superficial laminae after lesion rhizotomy and repair. A significant increase in glial reactivity was observed after lesion induction. However, neither human PRP gel application nor hESC engrafting exacerbated glial reactivity, in comparison to the DRZ group. These findings directly reflected in the functional recovery from these groups. Scale bar = 50 *μ*m.

**Figure 6:**
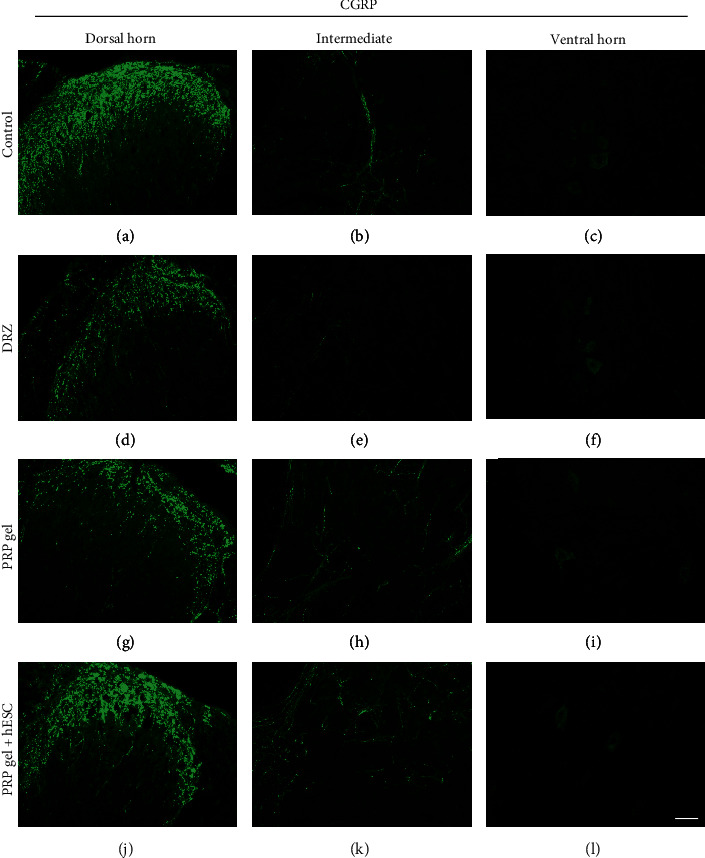
Representative immunofluorescence micrographs of CGRP immunolabeling on the ipsilateral side of the spinal cord after rhizotomy and repair, 1 week after lesion. Similar to VGLUT1 immunolabeling, a significant decrease in CGRP immunoreactivity was observed following DRZ. However, the “hESC” group showed a great improvement in CGRP-positive fibers in comparison to that of the DRZ group. Scale bar = 50 *μ*m.

Quantitative measurements of VGLUT1 (to detect glutamatergic synaptic changes), GFAP (to detect astroglial reactivity) and Iba-1 (to detect microglial reactivity) immunoreactivity in the spinal cord after rhizotomy and repair were carried out 1 (acute phase) week after lesion induction. Qualitative images of CGRP-positive fibers were also analyzed 1 week after lesion and repair. Representative immunofluorescence images of the lesioned side of the spinal cord are shown in Figures 4–6.

### 3.8. Glutamatergic Synapses

Intense VGLUT1 immunolabeling on the contralateral side was observed in all experimental animals, which was attributed to the large number of inputs to the contralateral neurons compared to those on the lesioned side. In contrast, the immunostaining showed lower synaptic density on the ipsilateral side in all experimental groups—except the control group (unlesioned)—confirming the outcomes of the surgical procedure (the loss of primary afferents), 1 week after lesion induction (Figure 4, Table 3). However, it was possible to observe an increase in punctate labeling, after root repair by PRP, which was statistically significant in the hESC group in comparison to the DRZ group. This is an important finding of the present study and suggests that root repair with the PRP gel enabled the reentry of fibers into the spinal cord and hESCs may enhance the regeneration of primary afferents.

### 3.9. Astrogliosis

Immunolabeling for GFAP demonstrated a significant increase in astrocyte activity after lesion induction, as demonstrated by the presence of GFAP-positive activated astrocytes in the spinal superficial dorsal laminae in all experimental groups—except for the control group (unlesioned), which showed a low reactivity for GFAP (Figures 5(a), 5(c), 5(e), and 5(g)). However, the astroglial reactivity was not significantly further increased after human PRP gel application or hESC engrafting, 1 week after lesion induction: control = 115.99 ± 9.04; DRZ = 678.65 ± 10.64; PRP gel = 701.15 ± 13.02; and PRP gel + hESCs = 680.02 ± 5.46. Relative immunoreactivity from the ipsilateral side is expressed as the mean ± standard error of the mean of the density of pixels; *n* = 5 per group.

### 3.10. Microgliosis

Similar results were observed in microglial reactivity. A significant increase in microglial activity after lesion induction was observed as evidenced by Iba-1 immunostaining in the spinal superficial dorsal laminae in all experimental groups, except for the control group (unlesioned), which showed low reactivity for Iba-1 (Figures 5(b), 5(d), 5(f), and 5(h)). Once again, the microglial reactivity was not significantly further increased after human PRP gel application or hESC engrafting, 1 week after lesion induction: control = 58.63 ± 12.75; DRZ = 445.19 ± 7.19; PRP gel = 451.37 ± 11.50; and PRP gel + hESCs = 443.52 ± 12.35. Relative immunoreactivity from the ipsilateral side is expressed as the mean ± standard error of the mean of the density of pixels; *n* = 5 per group. These are also important findings because the nonexacerbation of glial reactivity may have facilitated the penetration of regenerating axons into the spinal surface.

### 3.11. CGRP-Positive Fibers

There was a high density of fibers exhibiting CGRP immunoreactivity in the control group (unlesioned) in the dorsal horn of the spinal cord in laminae I and II, and there was a lower density of these fibers in lamina V. CGRP-like immunoreactivity was also localized in motoneurons of the ventral horn (Figure 6). However, compared to the control results, immunostaining showed lower immunoreactivity on the ipsilateral side after DRZ in all analyzed spinal laminae, especially in the superficial laminae. In contrast, an increase in CGRP-immunoreactivity was observed in the hESC group in comparison to that of the DRZ group. We emphasize that there was no increase in CGRP-immunoreactivity or ectopic expression in relation to the unlesioned animal, which corroborates the lack of observed hypersensitivity.

### 3.12. Transmission Electron Microscopy (TEM)

Through transmission electron microscopy (TEM), it was possible to investigate morphological and structural aspects of the dorsal root repair with the PRP gel, exploring the reconnected root and spinal microenvironment, 8 weeks after lesion induction when the molecular changes were confirmed.

**Figure 7:**
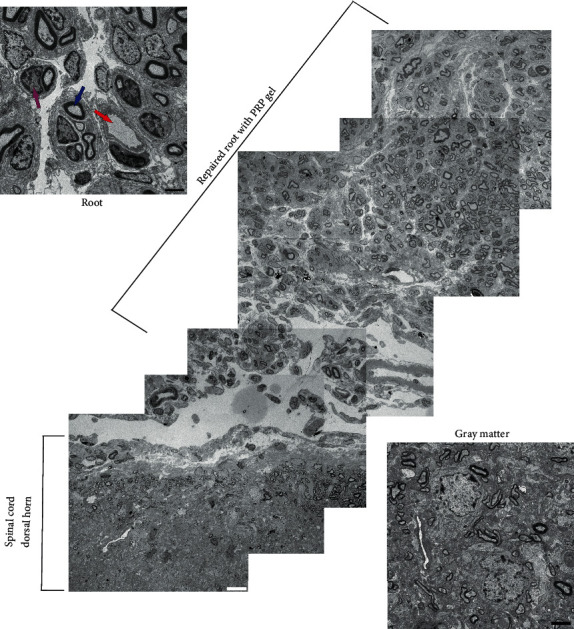
Representative transmission electronic micrographs of the repaired root and spinal cord dorsal horn (superficial region), 8 weeks after dorsal rhizotomy (DRZ) and repair with PRP gel. The extent of the repaired root can be observed with a significant number of myelinated axons (blue arrow), blood vessels (red arrow), and many Schwann cells (purple arrow). The image also highlights the subtle boundary between PNS/CNS clearly delimited by the dorsal root transition zone (DREZ), which contains central and peripheral nervous tissue. Moreover, proximal to the DREZ, myelin sheaths formed by oligodendrocytes can be observed, while further from the DREZ, the sheaths are formed by Schwann cells and wrapped the endoneurium. Their presence on the spinal surface of the root repaired in these morphological and structural conditions is one of the requirements for functional recovery.

As seen in Figure 7, which corresponds to an assembly of representative TEM photomicrographs, it is possible to observe the presence of the repaired dorsal root at the spinal surface of the lesion site, presenting a large number of myelinated axons (blue arrow) grouped into small fascicles, blood vessels (red arrow), and many Schwann cells (purple arrow)—being a strong indication of the repaired root's functionality, providing physical support for recovery.

## 4. Discussion

A reproducible model for investigating the regeneration of sensory pathways and the consequences of their absence is the dorsal rhizotomy (DRZ) at the lumbar level in rats, which is an important source of information for preclinical studies of injuries at the interface between the CNS and PNS [28]. The DRZ affects the central projections of the axons of sensory neurons present in the dorsal ganglia, disconnecting them from the spinal cord surface in the dorsal column and imparting partial or complete loss of sensitivity in the affected limb. These losses can be present for a long time or can even become permanent, and they were observed in the “DRZ” group in the morphological analysis and in the functional test.

As a result of the lesion, glutamatergic (VGLUT1) synapses are lost and reorganized, resulting in the disappearance of specific synaptic clusters [29]. Therefore, through different histological techniques, it was possible to assert that the experimental model used here resulted in the withdrawal of primary afferents. The evidence for this was the extensive degeneration in the dorsal column of the spinal cord and a concomitant decrease in immunoreactivity for VGLUT1, which mainly occurred in the superficial laminae and was accompanied by an intense reactivity of glial cells. Microglial reactivity is related to the removal of VGLUT1 synapses in the ventral horn, while such loss mechanism is microglia-independent for the dorsal horn [29]. These morphological changes were reflected in functional behavior, resulting in the loss of sensibility in the ipsilateral site to the lesion site. To reverse or minimize this scenario and to achieve functional recovery, the present study proposed the use of platelet-rich plasma (PRP) gel as an adhesive element for reconnecting the dorsal roots to the spinal cord surface (acting as a biological glue for structural repair) and as a scaffold for bioengineered human embryonic stem cells (hESCs). It is important to highlight that the right biological scaffold needs to provide structural integrity for cells. Therefore, the scaffold should be highly porous and should have pores of a suitable size because released factors are important for stem cell seeding, migration, and nutrient supply [30]. In this sense, the PRP scaffold proved to be suitable for such applications.

Currently, the use of platelet-rich plasma (PRP), in its liquid or gel form, extends to a wide range of surgical procedures, and there are several studies that have demonstrated the benefits of using PRP in treating a wide spectrum of injuries. Another platelet-rich blood derivative that has been used in regenerative medicine is Choukroun's platelet-rich fibrin [31]. PRF consists of a leukocyte-platelet-rich dense fibrin matrix with cytokines, platelets, and blood stem cells combined altogether [32]. This biomaterial can act as a proper scaffold for cells, and it is capable of releasing higher quantities of growth factors compared to PRP [33, 34], properties that permit the crescent use of both PRF and PRP in surgical procedures [33, 35].

Despite the good potential of PRF as a nerve guidance conduit [36], the reported benefits of PRF in nervous system injury treatment are still moderate [37, 38]. In the context of nervous system disorders, the benefits of PRP already described in recent literature involve neuroprotection and prevention of apoptosis, mediation of neuroinflammation, and stimulation of angiogenesis and axonal regeneration [39]. Additionally, it is important to note that the present study is aimed at analyzing the modified hESC contribution in DRZ in the absence of the cells commonly present in PRF.

In parallel, to enhance nerve regeneration and make it even more efficient, several studies have explored the potential of different cell therapy strategies for the treatment of a wide spectrum of diseases and injuries in the central nervous system (CNS) [11, 40, 41]. These cells have important characteristics that make them useful in the regenerative process, such as their capacity for self-renewal [40], as well as their angiogenic, antiapoptotic, anti-inflammatory, and immunomodulatory properties [12, 41]. These properties are enabled by the production of different trophic factors and cytokines [11], according to their respective origin (tissue source) and the stimulus they received. The stem cells can be used as progenitors of differentiated cells transplanted to injury sites (reposition therapy) or as a source of trophic molecules that contributes to tissue regeneration [40]. Nowadays, both applications have been explored in translational regenerative medicine especially due to a crescent understanding of molecular mechanisms related to therapeutic effects of these cells [42]. We focused on the stem cell therapy as a promising source of growth factors. In this context, we employed hESCs overexpressing FGF2, due to their accessibility to undergo genetic modifications while retaining the pluripotency [43].

Here, the PRP gel used in combination with cell therapy contributed to axon regeneration and the nonexacerbation of glial reactivity (which may have facilitated the penetration of regenerating axons) in the analyzed spinal cord laminae, as well as by the positive results in a functional test. We believe that these regenerative improvements have three causes: root repair itself, the intrinsic action of platelets, and the production of factors by hESCs, especially FGF2.

Regarding the first cause, the PRP gel allowed the physical reconnection of the lesioned root to the surface of the spinal cord, allowing the reentry of fibers. Therefore, a large number of axons were observed inside the repaired root, which contributed to the restoration of the reflex arc. It is known that root repair alone is neuroprotective and allows axonal regeneration [44–46]. Important studies have observed greater survival of motoneurons in cases of ventral root repair [47], while other studies [26] have observed improved recovery of primary fibers in cases using dorsal root repair. The authors suggest that the repaired root increases the production of neurotrophic factors and that the persistent opening of the blood-brain barrier (BBB) allows trophic substances to stimulate the regeneration of axons.

Secondly, the platelet biology itself may have favored the improvement of the lesioned microenvironment. Specifically, the PRP gel is biodegraded over time and a wide range of molecules are released by the platelets' granules. As an example, granules release cytokines, as well as trophic and growth factors, including platelet-derived growth factor (PDGF), transforming growth factor-*β* (TGF-*β*), vascular endothelial growth factor (VEGF), growth factor basic fibroblasts (bFGF), insulin-like growth factor (IGF-1), hepatocyte growth factor (HGF), and epidermal growth factor (EGF) [48, 49]. These molecules can even regulate the biological effects of other growth factors such as nerve growth factor (NGF) and brain-derived neurotrophic factor (BDNF) [20]. Together, these factors play a significant role in tissue regeneration, regulating and mediating proliferation, allowing cell differentiation stimulating angiogenesis, and neuroprotection [48].

Lastly, it is also known that trophic factors produced by stem cells (such as BDNF and GDNF by bioengineered hESCs) mediate survival, differentiation, growth of neurites, and functional plasticity in the central and peripheral nervous system. In addition, they play a crucial role in maintaining the specific functions of distinct populations of neurons, not only in development, but also after spinal cord and nerve injuries [15, 50]. In addition, FGF2 overexpression by bioengineered hESCs may facilitate neuronal repair, since several experimental studies in the CNS and PNS have revealed that this growth factor plays an important role in neuronal axonal regeneration and repair after brain injuries [51] and spinal cord injuries [14, 15].

In spinal cord injuries, functional recovery is attributed to FGF-mediated promotion of neuronal survival [52–54], reduction of glial reactivity [14], and stimulation of angiogenesis [55], which results in a reduction in the lesion volume [56]. FGF2 is also capable of stimulating synaptic plasticity [57–59]. Most likely, after cell engraftment, the expression of trophic factors increases, due to stimulation of the microenvironment, which may be responsible for the regenerative improvements observed in the hESC group.

Nevertheless, it is important to highlight that the therapeutic approach used herein seems to allow oriented axonal regeneration, since there was no aberrant sprouting of regenerated fibers, and ectopic expression of VGLUT1 and CGRP. Thus, we could not detect signs of allodynia nor hyperalgesia.

## 5. Conclusion

Overall, the results of the present study indicate that the dorsal root repair performed with the support of platelet-rich plasma (PRP) gel contributed to axonal regeneration after dorsal rhizotomy, as seen by the histological and functional observations. In addition, the bioengineered human embryonic stem cell (hESC) therapy further enhanced the axonal regeneration process after root repair. Therefore, our data support the idea that early root reconnection, combined with the engrafting of bioengineered stem cells is effective, opening new possibilities in translational medicine.

## Figures and Tables

**Table 1:** TaqMan assays used in the qRT-PCR analysis.

Genes	Code
GAPDH	Hs04420632_g1
FGF2	Hs00266645_m1
BDNF	Hs02718934_s1
GDNF	Hs01931883_s1

**Table 2:** Antibodies used in the immunofluorescence assay.

Target	Host	Conjugate	Dilution	Manufacturer	Cat. code
VGLUT1	Rabbit	—	1/1000	Synaptic systems	135303
GFAP	Rabbit	—	1/1500	Abcam	AB7260
Iba-1	Rabbit	—	1/750	Wako	019-19741
CGRP	Rabbit	—	1/1000	Sigma	C8198
Rabbit IgG	Donkey	CY2	1/500	Jackson	711-225-152
Rabbit IgG	Donkey	CY3	1/500	Jackson	711-165-152

**Table 3:** Immunoreactivity related to synaptic vesicles containing the vesicular glutamate transporter type 1 (VGLUT1) (ipsi/contralateral ratio) was obtained by measuring the integrated density of pixels in the spinal cord in III, V, and VI and IX laminae, 1 week after rhizotomy and repair of dorsal nerve roots (mean ± standard error of mean).

Laminae	Control	DRZ	PRP gel	hESCs + PRP gel
III	1.08 ± 0.15	0.23 ± 0.11	0.40 ± 0.12	0.54 ± 0.15
V and VI	1.11 ± 0.15	0.13 ± 0.07	0.29 ± 0.11	0.35 ± 0.08
IX	1.03 ± 0.10	0.10 ± 0.05	0.23 ± 0.09	0.27 ± 0.13

## Data Availability

Data are available on request.

## Abbreviations

BBB: Blood-brain barrier
BDNF: Brain-derived neurotrophic factor
bFGF: Basic fibroblast growth factor
cDNA: Complementary deoxyribonucleic acid
CEMIB: Multidisciplinary Center for Biological Investigation
CGRP: Calcitonin gene-related peptide
CNS: Central nervous system
CONCEA: National Council of Animal Experimentation
DAPI: 4′,6-Diamidine-2′-phenylindole dihydrochloride
DOX: Doxycycline
DRZ: Dorsal rhizotomy
EGF: Epidermal growth factor
FGF2: Fibroblast growth factor 2
GAPDH: Glyceraldehyde 3-phosphate dehydrogenase
GDNF: Glial cell line-derived neurotrophic factor
GFP: Green fluorescent protein
GFAP: Glial fibrillary acidic protein
GDNF: Glial-derived neurotrophic factor
hESCs: Human embryonic stem cells
HGF: Hepatocyte growth factor
Iba1: Ionized calcium binding adaptor molecule 1
IDP: Integrated density of pixels
IGF-1: Insulin-like growth factor
mRNA: Messenger ribonucleic acid
NGF: Nerve growth factor
Oct3/4: Octamer-binding transcription factor 4
PB: Phosphate buffer
PBS: Saline phosphate buffer
PDGF: Platelet-derived growth factor
PRF: Platelet-rich fibrin
PRP: Platelet-rich plasma
PNS: Peripheral nervous system
qRT-PCR: Quantitative reverse transcription polymerase chain reaction
SEM: Standard error of the mean
SSEA-1: Stage-specific embryonic antigen-1
SSEA-4: Stage-specific embryonic antigen-4
TEM: Transmission electron microscopy
TGF-*β*: Transforming growth factor-*β*
UNICAMP: University of Campinas
VEGF: Vascular endothelial growth factor
VGLUT1: Vesicular glutamate transporter 1.

## Additional Points

*Highlights*. (1) Spinal cord dorsal rhizotomy (DRZ) can be repaired with platelet-rich plasma (PRP) gel. (2) PRP gel treatment reduced the degenerative effects of DRZ. (3) PRP and human embryonic stem cells (hESCs) upregulate proregenerative molecules. (4) hESCs combined with PRP gel scaffolds promoted substantial axonal regeneration within the dorsal horn

## Supplementary Materials

Supplementary Materials Methodological details for obtaining PRP from human blood and characterizing the pluripotency of bioengineered hESCs by flow cytometry.
