# Supplementary figures and images for: Spinal Reflex Recovery after Dorsal Rhizotomy and Repair with Platelet-Rich Plasma (PRP) Gel Combined with Bioengineered Human Embryonic Stem Cells (hESCs)
Source: Stem Cells Int. 2020 Oct 29;2020:8834360. doi: 10.1155/2020/8834360 (PMC7647752; doi:10.1155/2020/8834360)

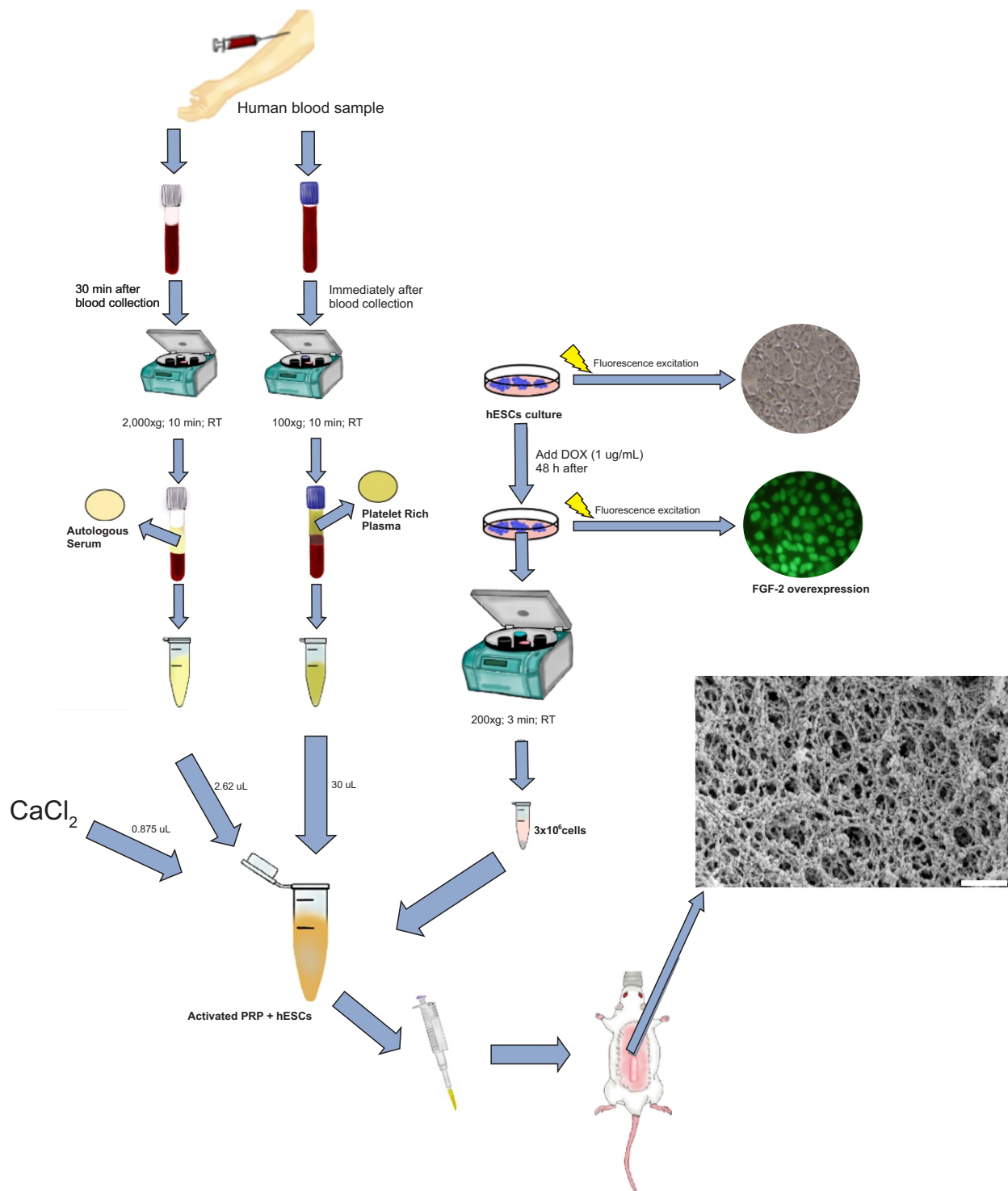

Supplement: Supplementary Materials — Methodological details for obtaining PRP from human blood and characterizing the pluripotency of bioengineered hESCs by flow cytometry. [file 8834360.f1.zip › Figure S1.pdf]

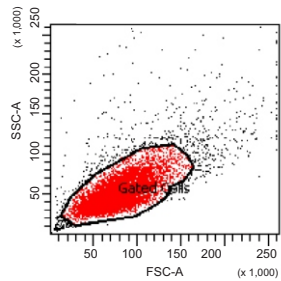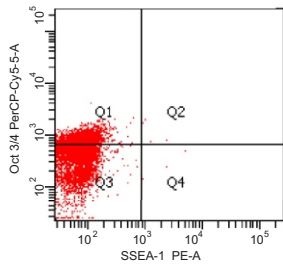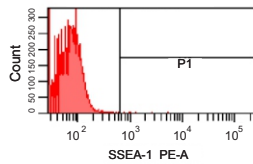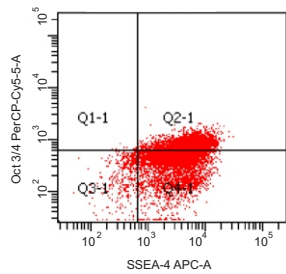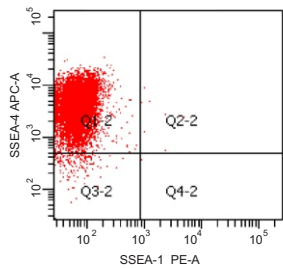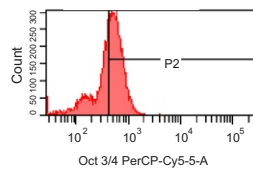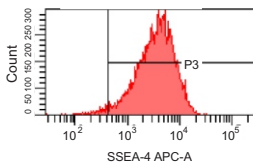

| Population    | #Events | %Parent | %Total |
|---------------|---------|---------|--------|
| All Events    | 10,000  | ####    | 100.0  |
| Gated Cells   | 9,253   | 92.5    | 92.5   |
| P1 (SSEA-1)   | 12      | 0.1     | 0.1    |
| P2 (Oct 3/4 ) | 5,609   | 60.6    | 56.1   |
| P3 (SSEA-4)   | 9,063   | 97.9    | 90.6   |

Supplement: Supplementary Materials — Methodological details for obtaining PRP from human blood and characterizing the pluripotency of bioengineered hESCs by flow cytometry. [file 8834360.f1.zip › Figure S2.pdf]

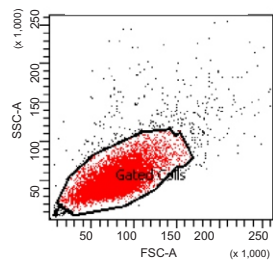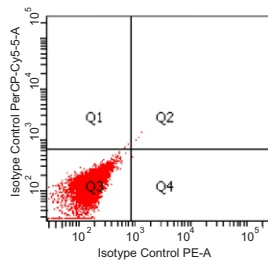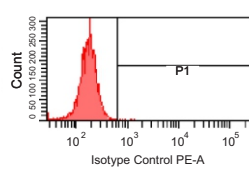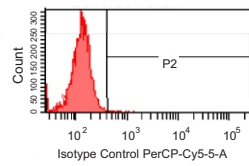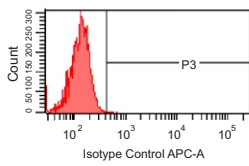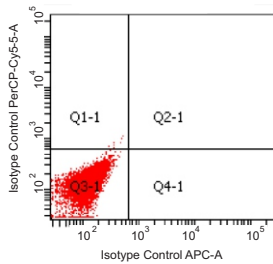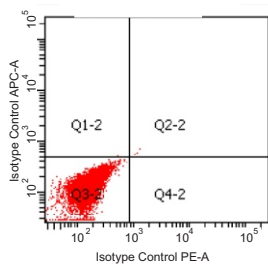

| Population  | #Events | %Parent | %Total |
|-------------|---------|---------|--------|
| All Events  | 10,000  | ####    | 100.0  |
| Gated Cells | 9,518   | 95.2    | 95.2   |
| P1 (PE-A )  | 9       | 0.1     | 0.1    |
| P2 (PerCP ) | 25      | 0.3     | 0.2    |
| P3 (APC-A)  | 7       | 0.1     | 0.1    |

Supplement: Supplementary Materials — Methodological details for obtaining PRP from human blood and characterizing the pluripotency of bioengineered hESCs by flow cytometry. [file 8834360.f1.zip › Figure S3.pdf]
